# Exploring the association of mechanical power with mortality and phenotypes among patients with acute respiratory distress syndrome: a retrospective analysis

**DOI:** 10.3389/fmed.2026.1705637

**Published:** 2026-03-13

**Authors:** Qi Zhang, Na Liu, Fan Wang, Huiyong Wang, Renshuang Ding, Yan Li, Zhiyong Wang, Yan Li, Mingxing Fang

**Affiliations:** 1 Department of Critical Care Medicine, Hebei Medical University Third Hospital, Hebei, China; 2 Critical Disease Data Analysis and Intelligent Diagnosis and Treatment Engineering Research Center of Hebei Province, Hebei Medical University Third Hospital, Hebei, China; 3 Department of Emergency, The Fourth Hospital of Hebei Medical University, Hebei, China; 4 School of Public Health, Hebei Medical University, Hebei, China; 5 School of Information Science and Engineering, Hebei University of Science and Technology, Hebei, China; 6 Department of Critical Care Medicine, The Second Affiliated Hospital of Xi'an Medical University, Shanxi, China; 7 Department of Emergency and Internal Medicine, The People's Hospital of Tiemenguan, Tiemenguan, China

**Keywords:** acute respiratory distress syndrome, consensus clustering analysis, mechanical power, mechanical ventilation, mortality

## Abstract

**Introduction:**

Mechanical power (MP) quantifies the energy delivered from a ventilator to the respiratory system and is a key contributor to ventilator-induced lung injury. This study evaluated the association between MP and mortality in patients with acute respiratory distress syndrome (ARDS), and examined whether this relationship differs across data-driven ARDS phenotypes.

**Methods:**

Patients with ARDS requiring invasive ventilation were identified from the MIMIC-IV database. The association between MP and mortality was assessed using logistic regression, Kaplan–Meier survival analysis, and Cox proportional hazards models. The optimal MP threshold was determined using maximally selected rank statistics. Unsupervised clustering was used to identify ARDS phenotypes and evaluate phenotype-specific responses to MP.

**Results:**

A total of 1,333 patients were included. An MP < 18.7 J/min was associated with significantly lower 28-day mortality. Among MP components, the elastic-dynamic component showed the strongest association with mortality; the elastic-static component had a weaker association, and the resistive component was not significant. Respiratory rate was the strongest predictor of mortality. Three phenotypes were identified. Phenotype I (mechanical stress-dominant): poor respiratory mechanics and the highest mortality. Phenotype II (oxygenation-preserved with mild inflammation): better oxygenation, less organ dysfunction, and the lowest mortality. Phenotype III (systemic hyperinflammation with metabolic dysregulation): significant laboratory abnormalities, strong association with high MP, and increased mortality.

**Discussion:**

High mechanical power (MP) was independently associated with increased mortality in patients with ARDS. An MP threshold of 18.7 J/min demonstrated prognostic relevance for mortality risk stratification, and the association between MP and outcomes varied across ARDS phenotypes, highlighting the potential value of phenotype-informed ventilation strategies.

## Introduction

Acute respiratory distress syndrome (ARDS) is an acute diffuse inflammatory lung injury caused by various insults, characterized by damage to the alveolar epithelium and pulmonary capillary endothelium, increased vascular permeability, pulmonary edema, atelectasis, and reduced functional lung volume. These pathophysiological changes lead to increased intrapulmonary shunt and dead space, accompanied by inflammation, hyaline membrane formation, and alveolar hemorrhage (1, 2). Despite advances in supportive care, ARDS remains a major global health burden, with reported mortality rates ranging from 30% to 45% (3). Mechanical ventilation is the cornerstone of supportive therapy for ARDS, as it improves oxygenation and reduces the work of breathing. However, inappropriate ventilatory settings may aggravate ventilator-induced lung injury (VILI) by excessive mechanical stress and strain on vulnerable lung units (4). Therefore, identifying ventilatory parameters that better capture the injurious potential of mechanical ventilation is critical for optimizing lung-protective strategies.

Mechanical power (MP), which integrates tidal volume, respiratory rate, and driving pressure, has emerged as a comprehensive measure of the total mechanical power delivered from the ventilator to the respiratory system and is increasingly recognized as a key determinant of VILI (5). Previous studies have demonstrated a strong association between elevated MP and adverse outcomes. Serpa et al. (6) reported that mechanical power exceeding 17.0 J/min was independently associated with increased ICU and 30-day mortality in critically ill patients from the MIMIC-III (Medical Information Mart for Intensive Care-III) and eICU database (eICU Collaborative Research Database). Similarly, Zhang et al. (7) showed that ideal body weight–standardized mechanical power provided superior prognostic performance compared with absolute mechanical power. Collectively, these findings underscore the clinical relevance of limiting excessive mechanical power during ventilation. Despite these insights, several important gaps remain. Most existing evidence is derived from retrospective analyses, and the relative contributions of individual mechanical power components to patient outcomes are not fully understood (8, 9). Moreover, ARDS is a heterogeneous syndrome, and it remains unclear whether the association between MP and prognosis is consistent across different clinical and physiological phenotypes.

Therefore, the primary aim of this study was to evaluate the association between mechanical power and short-term mortality in invasively ventilated patients with ARDS using data from the MIMIC-IV database. The secondary objectives were to assess the prognostic contribution of individual mechanical power components and to explore whether distinct ARDS phenotypes modify the relationship between mechanical power and clinical outcomes.

## Methods

### Study design

This study was a retrospective analysis based on the MIMIC-IV (Medical Information Mart for Intensive Care-IV) database, which included data from patients with acute respiratory distress syndrome treated in the intensive care unit at Beth Israel Deaconess Medical Center from 2008 to 2019. One author completed the courses and tests of the Collaborating Institution Training Program for “Data or Specimen Studies” and obtained a license to use the MIMIC database (Number: 9535772). Because the patient privacy information in the database has been anonymized, this study does not require ethical approval.

### Patient selection

Patients in the MIMIC-IV database who were older than 18 years, received invasive mechanical ventilation for more than 24 h, and met the 2012 Berlin definition of ARDS were included (10). The Berlin definition was operationalized as “bilateral infiltrates on chest imaging, a P/F ratio < 300 mmHg with PEEP ≥ 5 cmH_2_O, and absence of heart failure.” Bilateral infiltrates were identified by screening chest radiology reports for the presence of keywords such as “edema” or the combination of “bilateral” and “infiltrates” in the free-text notes, following previously published approaches (11, 12). Heart failure was identified via ICD-9/ICD-10 codes. Patients without sufficient ventilatory variables to calculate mechanical power were excluded. For patients with multiple ICU admissions, only the first admission was included for analysis.

### Variable extraction

The collected data included the following: (1) Demographic characteristics: age, height, weight, and body mass index (BMI); (2) Severity scores: sequential organ failure assessment (SOFA score) and acute physiology score-III (APS-III); (3) Vital signs: systolic blood pressure (ABPs), diastolic blood pressure (ABPd), and heart rate; (4) Respiratory characteristics: tidal volume, spontaneous respiratory rate, total respiratory rate, set respiratory rate, PEEP, plateau pressure, peak airway pressure, and mechanical ventilation time; (5) Laboratory variables: white blood cell (WBC), platelet (PLT), blood urea nitrogen (BUN), blood glucose, creatinine, and lactate (Lac) levels; (6) Blood gas analysis parameters: oxygenation index on the first day (P/F ratio), arterial pH value, arterial partial pressure of oxygen (PaO_2_), arterial partial pressure of carbon dioxide (PaCO_2_), and HCO_3_-; and (7) Outcomes: 28-day mortality, ICU mortality, in-hospital mortality, and length of ICU stay. All the data were extracted via the Google Cloud BigQuery and Structured Query Language (SQL) in PostgreSQL (version 12.0). Indicators with more than 35% of the total values missing were excluded. The outliers were defined as values outside the 1.5-fold interquartile range. After excluding outliers, all variables were collected at the time of ARDS diagnosis within the first 24 h to ensure that the variables used in the prediction and association analyses were reflective of the initial disease severity and not confounded by later interventions or disease progression. The miss forest model was then employed to impute missing values (Additional file: Figure S1).

### Mechanical power

Mechanical power (MP) was calculated using the mean values of key ventilatory variables collected during the first 24 h after ARDS diagnosis, rather than arbitrarily selected or extreme (worst) values, including tidal volume, respiratory rate, driving pressure, and peak inspiratory pressure. To ensure the physiological validity of the mechanical power calculation, only measurements recorded during volume-controlled ventilation were included. This averaging strategy was adopted to reflect the overall ventilatory energy exposure during the early phase of ARDS, while minimizing the influence of short-term fluctuations related to transient clinical interventions. Plateau pressure values were obtained exclusively during volume-controlled ventilation, allowing appropriate application of the simplified Gattinoni (8) equation in a retrospective context. Mechanical power was computed using the standard formula: MP = 0.098 × RR × Vt × (Ppeak – $\frac{1}{2}$ × DP) (13). Driving pressure was calculated as the difference between plateau pressure and positive end-expiratory pressure (DP = Pplat – PEEP) (14). We further decomposed mechanical power into three components to evaluate the association of different mechanical components on mortality, using a methodology similar to that described by Costa et al. (15).

Elastic-static component: 0.098^*^VT^*^RR^*^PEEP

Elastic-dynamic component: 0.098^*^VT^*^RR^*^0.5^*^DP (DP = Pplat – PEEP)

Resistive component: 0.098^*^VT^*^RR^*^(Ppeak – Pplat).

### Statistical analysis

All statistical analyses were performed using R software (version 4.3.1). The primary objective was to evaluate the association between mechanical power (MP) and mortality in patients with ARDS. Accordingly, univariate logistic regression and Cox proportional hazards models were used to assess the associations between MP and ICU mortality, in-hospital mortality, and 28-day mortality. Kaplan–Meier survival curves were constructed, and differences between groups were compared using the log-rank test. A two-sided *P* value < 0.05 was considered statistically significant.

The optimal cutoff value for mechanical power in survival analysis was determined using maximally selected rank statistics implemented in the surv_cutpoint() function of the survminer package. Based on this cutoff, patients were categorized into high- and low-MP groups. Continuous variables were compared using Student's *t*-test or the Wilcoxon rank-sum test, as appropriate, and categorical variables were compared using the χ^2^ test. Normality was assessed using the Shapiro–Wilk test. Data are presented as mean ± standard deviation or median (interquartile range), as appropriate.

To reduce confounding, propensity score matching (PSM) was performed using a logistic regression model including age, BMI, disease severity scores (APS-III and SOFA), vital signs, laboratory variables, and arterial blood gas parameters. A 1:1 nearest-neighbor matching algorithm with a caliper width of 0.05 was applied. Covariate balance after matching was assessed using standardized mean differences (SMD), with values < 0.1 indicating adequate balance.

### Cluster analysis

Variables with right-skewed distributions were log-transformed to stabilize variance and subsequently standardized using z-score normalization to ensure comparability across variables. To explore clinical heterogeneity among ARDS patients, consensus clustering (CC) was performed using K-means as the base algorithm. The clustering procedure involved 80% subsampling, 1,000 iterations, and evaluation of candidate cluster numbers ranging from k = 2 to 6.

The optimal number of clusters was determined by jointly assessing cumulative distribution function (CDF) plots, delta area curves, and consensus matrices. Cluster stability was further evaluated using intracluster consistency scores, defined as the mean consensus value of all sample pairs within the same cluster, with values closer to 1 indicating greater within-cluster stability.

In addition, the proportion of ambiguously clustered pairs (PAC) was calculated to quantify the degree of uncertainty in cluster assignment. PAC reflects the proportion of sample pairs with intermediate consensus values, with lower values indicating more robust and reproducible clustering results. Based on these complementary metrics, a three-cluster solution demonstrated the most stable and reliable structure and was therefore selected for subsequent analyses.

To facilitate visualization of the high-dimensional clustering results, t-distributed stochastic neighbor embedding (t-SNE) was applied. As a non-linear dimensionality reduction technique, t-SNE was used solely for visualization purposes rather than for clustering itself, allowing intuitive representation of the separation among identified phenotypes, together with line plots (16, 17).

Following phenotype assignment, clinical characteristics and outcomes were compared across phenotypes. Continuous variables are presented as mean ± standard deviation or median (interquartile range), and categorical variables as percentages. Between-group comparisons were performed using the χ^2^ test, one-way ANOVA, or the Wilcoxon rank-sum test, as appropriate. All analyses were conducted using R software (version 4.3.1).

### External validation

To evaluate the external generalizability of the association between mechanical power (MP) and mortality, an external validation cohort was constructed using data from the Intensive Care Unit of the Third Hospital of Hebei Medical University. This cohort included adult patients (≥18 years) admitted between January 2023 and June 2025 who met the 2012 Berlin definition of ARDS and received invasive mechanical ventilation for more than 24 h. The same inclusion and exclusion criteria and variable definitions as those used in the derivation (MIMIC-IV) cohort were applied.

For external validation, ventilatory variables collected within the first 24 h after ARDS diagnosis were used to calculate MP and its individual components using identical formulas to those applied in the MIMIC-IV cohort. Missing data were imputed using the missForest algorithm.

To assess the prognostic relevance of MP in the external cohort, logistic regression was performed with in-hospital mortality as the primary outcome. Cox proportional hazards models and Kaplan–Meier survival analyses were subsequently conducted to examine the association between MP and in-hospital survival. Patients were further stratified according to the predefined MP cutoff value of 18.7 J/min derived from the MIMIC-IV cohort, which was directly applied to evaluate the discriminatory performance and robustness of this threshold in an independent population.

## Results

### Patients

**Figure 1:**
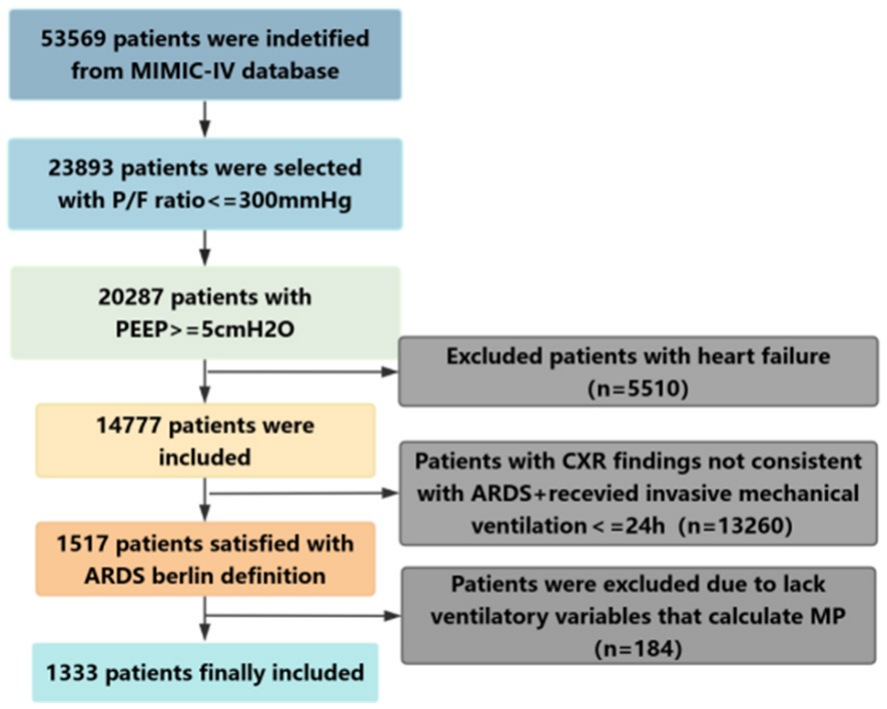
Flow chart of the inclusion and grouping of the study population. P/F ratio, PaO_2_/FiO_2_ ratio; PEEP, positive end-expiratory pressure; MP, mechanical power; ARDS, acute respiratory distress syndrome.

The MIMIC-IV database includes 53,569 ICU admissions. After excluding patients who received invasive mechanical ventilation for less than 24 h, 1,517 patients met the Berlin definition criteria for ARDS (2012). Among these, 184 patients were further excluded due to missing variables required for mechanical power (MP) calculation, resulting in a final cohort of 1,333 patients. The study flowchart is presented in Figure 1.

### Association between mechanical power and mortality

**Figure 2:**
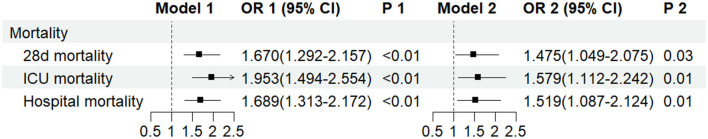
Association between mechanical power (MP) and mortality in the original cohort and matched cohort, stratified by the cutoff value of 18.7 J/min. Patients were categorized into low MP (≤18.7 J/min) and high MP (>18.7 J/min) groups. Odds ratios (ORs) and 95% confidence intervals (CIs) for 28-day, ICU, and in-hospital mortality are shown for the original cohort (Model 1) and the matched cohort (Model 2). OR, odds ratio; CI, confidence interval; MP, mechanical power.

**Figure 3:**
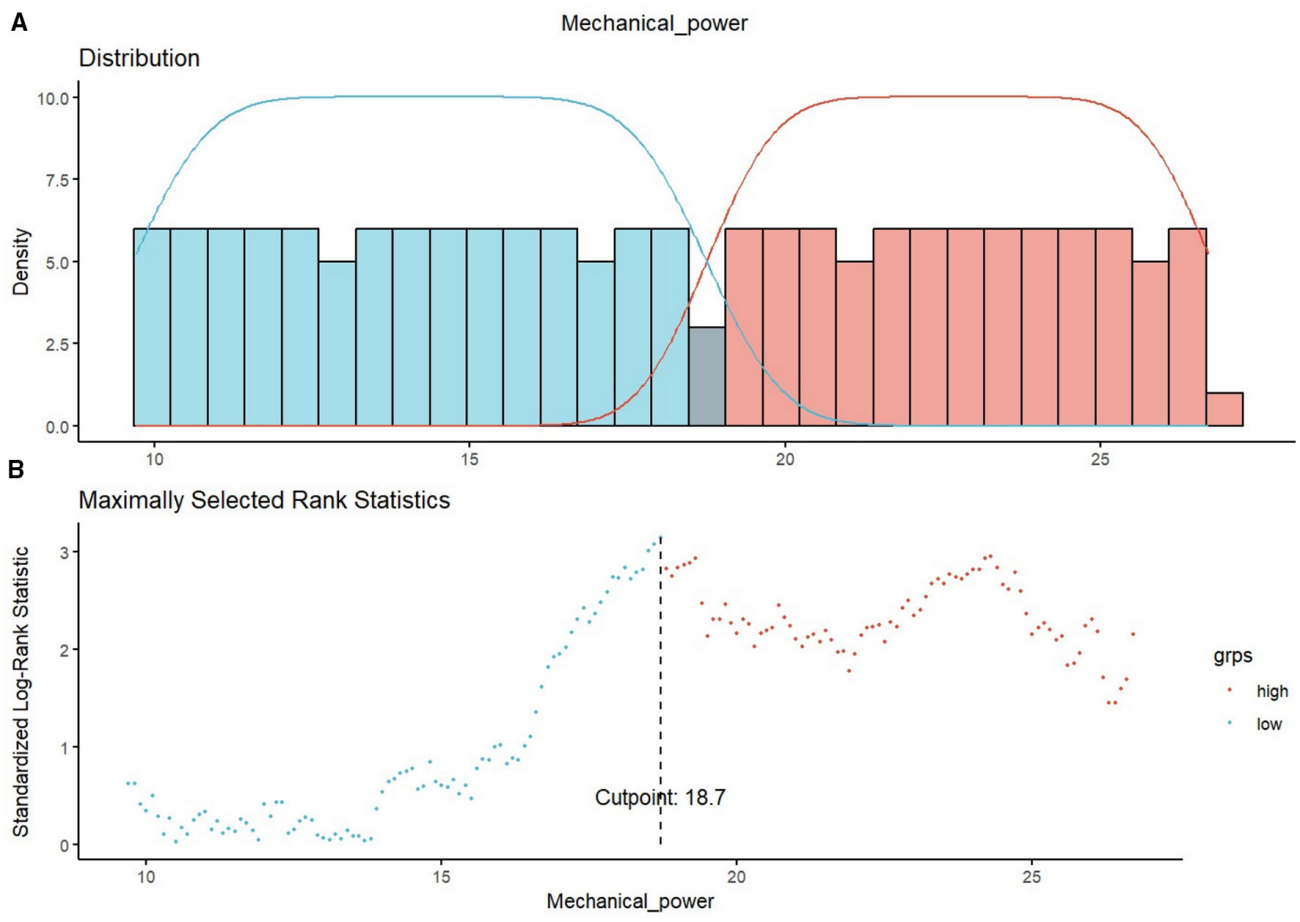
Distribution of the mechanical power and cutoff determination via maximally selected rank statistics. **(A)** The distribution of MP is shown for two groups: low MP (blue) and high MP (red). The histogram provides a visualization of the MP variable. **(B)** Maximally selected rank statistics were used to identify the cutoff for MP. The cutoff of 18.7 J/min is indicated by the vertical dashed line, which separates the low- and high-MP groups. This cutoff was selected on the basis of its ability to maximize the difference in survival outcomes between the two groups. MP, mechanical power.

During the first 24 h of mechanical ventilation, higher mechanical power was significantly associated with increased ICU mortality, in-hospital mortality, and 28-day mortality in the original cohort (Figure 2, Model 1). Using maximally selected rank statistics, an optimal MP cutoff of 18.7 J/min was identified (Figure 3). Patients were therefore categorized into high-MP and low-MP groups.

Before matching, significant differences were observed between groups in age, BMI, disease severity scores, vital signs, laboratory indices, and respiratory mechanics (Table 1 and Additional File Table S1). After 1:1 propensity score matching, 696 patients (348 per group) were included. Overall, covariate balance was substantially improved after matching, as assessed by standardized mean differences (Additional File Figure S2). However, a small but statistically significant difference in BMI remained between groups. Given the known clinical relevance of BMI in ARDS, we therefore performed a sensitivity analysis excluding patients with high BMI to further evaluate the robustness of the association between mechanical power and outcomes.

**Table 1:** Comparison of the covariates between the low MP group and high MP group in original cohort and matched cohort.

	**Original cohort**			**Matched cohort**		
	**MP** ≤ **18.7 J/min**	**MP** > **18.7 J/min**	***p***	**MP** ≤ **18.7 J/min**	**MP** > **18.7 J/min**	***p***
*N*	832	501		348	348	
**Demographic characteristics**						
Age [median (IQR)]	65.70 [55.00, 76.62]	59.10 [48.20, 68.70]	< 0.001	61.95 [50.58, 70.43]	61.95 [51.60, 70.70]	0.843
BMI [median (IQR)]	27.80 [24.10, 31.52]	30.90 [26.30, 35.30]	< 0.001	28.80 [24.90, 33.90]	30.20 [26.20, 33.80]	0.023
**The severity of illness**						
APSIII [median (IQR)]	67.00 [49.00, 86.00]	81.00 [59.00, 105.00]	< 0.001	75.00 [56.75, 94.00]	72.00 [54.75, 95.25]	0.859
SOFA score [median (IQR)]	6.90 [5.00, 9.10]	8.20 [6.20, 11.00]	< 0.001	8.00 [5.80, 10.10]	7.40 [5.60, 10.15]	0.394
**Vital signs**						
ABPd [median (IQR)]	57.45 [53.98, 61.00]	58.40 [54.60, 62.90]	0.009	58.00 [54.38, 62.00]	58.05 [54.75, 62.40]	0.890
ABPs [median (IQR)]	112.40 [107.00, 119.00]	108.70 [103.20, 115.00]	< 0.001	110.30 [105.40, 116.00]	109.10 [104.60, 116.00]	0.364
Heart rate [median (IQR)]	84.90 [73.88, 96.50]	92.60 [78.30, 105.00]	< 0.001	89.70 [77.05, 102.15]	88.55 [75.57, 100.30]	0.316
**Blood gas analysis parameters**						
Arterial PH [median (IQR)]	7.40 [7.30, 7.40]	7.30 [7.30, 7.40]	< 0.001	7.30 [7.30, 7.40]	7.30 [7.30, 7.40]	0.690
PaO_2_ [median (IQR)]	113.85 [98.22, 133.83]	105.30 [89.00, 121.70]	< 0.001	108.25 [93.30, 127.00]	108.70 [92.80, 125.73]	0.905
PaCO_2_ [median (IQR)]	39.30 [35.00, 43.00]	40.80 [36.50, 47.00]	< 0.001	40.00 [36.10, 43.42]	40.10 [35.88, 45.00]	0.871
HCO_3_ [median (IQR)]	22.00 [19.50, 24.72]	21.00 [17.80, 24.50]	< 0.001	21.50 [19.00, 24.20]	21.65 [18.00, 24.80]	0.818
P/F ratio [median (IQR)]	213.90 [160.75, 270.00]	170.30 [127.80, 221.20]	< 0.001	182.75 [139.07, 243.88]	190.00 [149.57, 238.95]	0.416
**Outcomes**						
Mechanical ventilation hour [median (IQR)]	54.35 [4.74, 94.55]	80.00 [47.00, 133.70]	< 0.001	61.75 [37.18, 113.85]	68.00 [41.75, 107.00]	0.344
ICU staydays [median (IQR)]	7.98 [4.74, 13.68]	9.85 [5.65, 15.83]	0.001	8.07 [5.33, 13.59]	8.28 [4.85, 13.96]	0.584
ICU mortality (%)	137 (16.47)	139 (27.74)	< 0.001	70 (20.11)	99 (28.45)	0.013
28-day mortality (%)	167 (20.07)	148 (29.54)	< 0.001	78 (22.41)	104 (29.89)	0.031
Hospital mortality (%)	177 (21.27)	157 (31.34)	< 0.001	82 (23.56)	111 (31.90)	0.018

In the matched cohort, elevated MP remained independently associated with higher ICU mortality, in-hospital mortality, and 28-day mortality (Figure 2, Model 2), confirming the robustness of the association between mechanical power and adverse outcomes.

### Contributions of mechanical power components

Among the three components of mechanical power, the elastic–dynamic component demonstrated the strongest association with 28-day mortality (OR = 1.267, 95% CI: 1.121–1.431; *P* < 0.01), whereas the elastic–static component showed a weaker association and the resistive component was not significantly associated with mortality (Additional File Figure S3). When individual ventilatory variables were evaluated, all parameters were standardized, and odds ratios reflected a one–standard deviation increase. Respiratory rate emerged as the strongest predictor of mortality (Additional File Figure S4). The composite index 4 × driving pressure + respiratory rate also remained significantly associated with 28-day mortality (OR = 1.286, 95% CI: 1.135–1.457; *P* < 0.01; Additional File Figure S5).

### Identification of ARDS phenotypes

**Figure 4:**
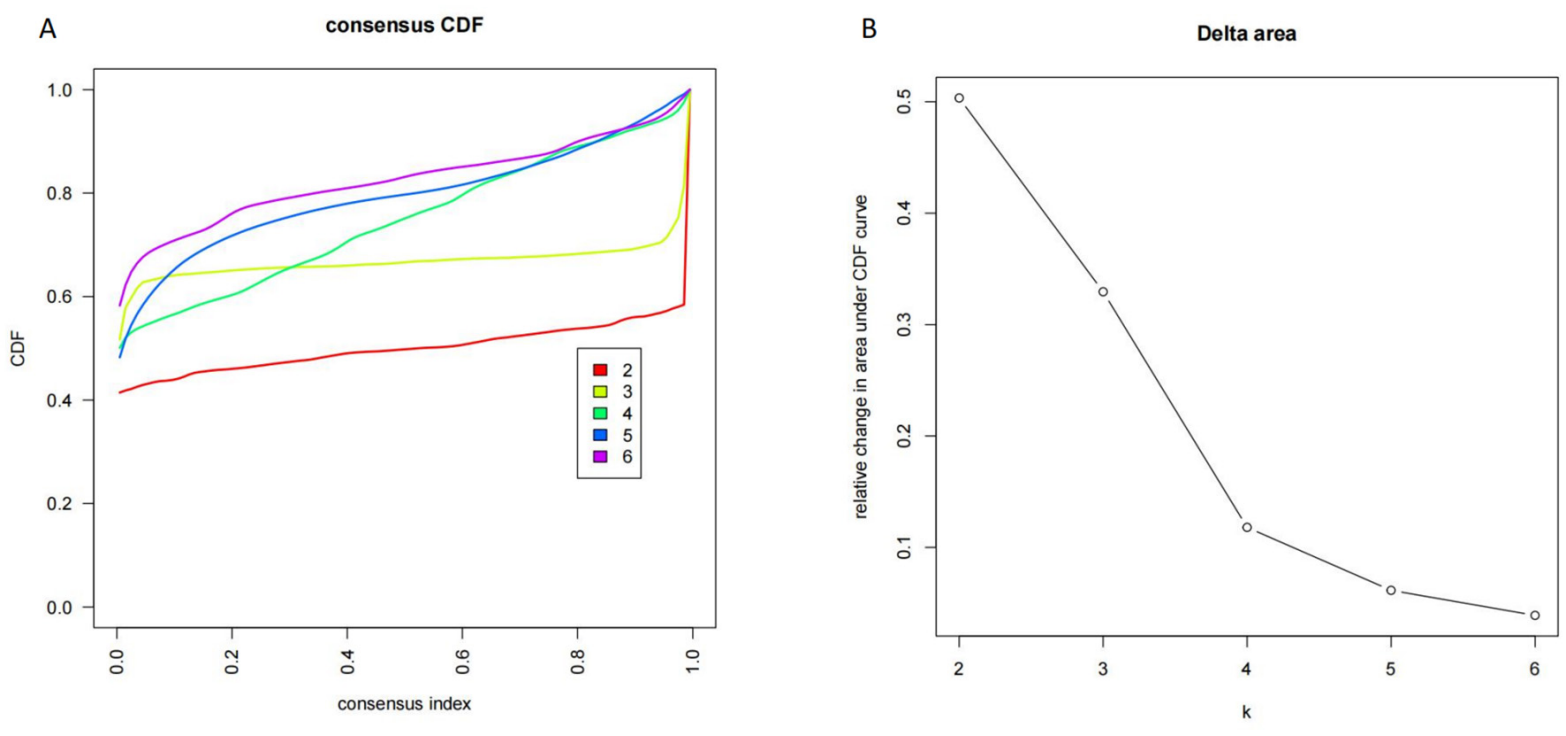
**(A)** Consensus cumulative distribution function (CDF) distributions with different cluster numbers k from 2 to 6. **(B)** Delta area of the consensus CDF for the number of clusters from 2 to 6.

**Figure 5:**
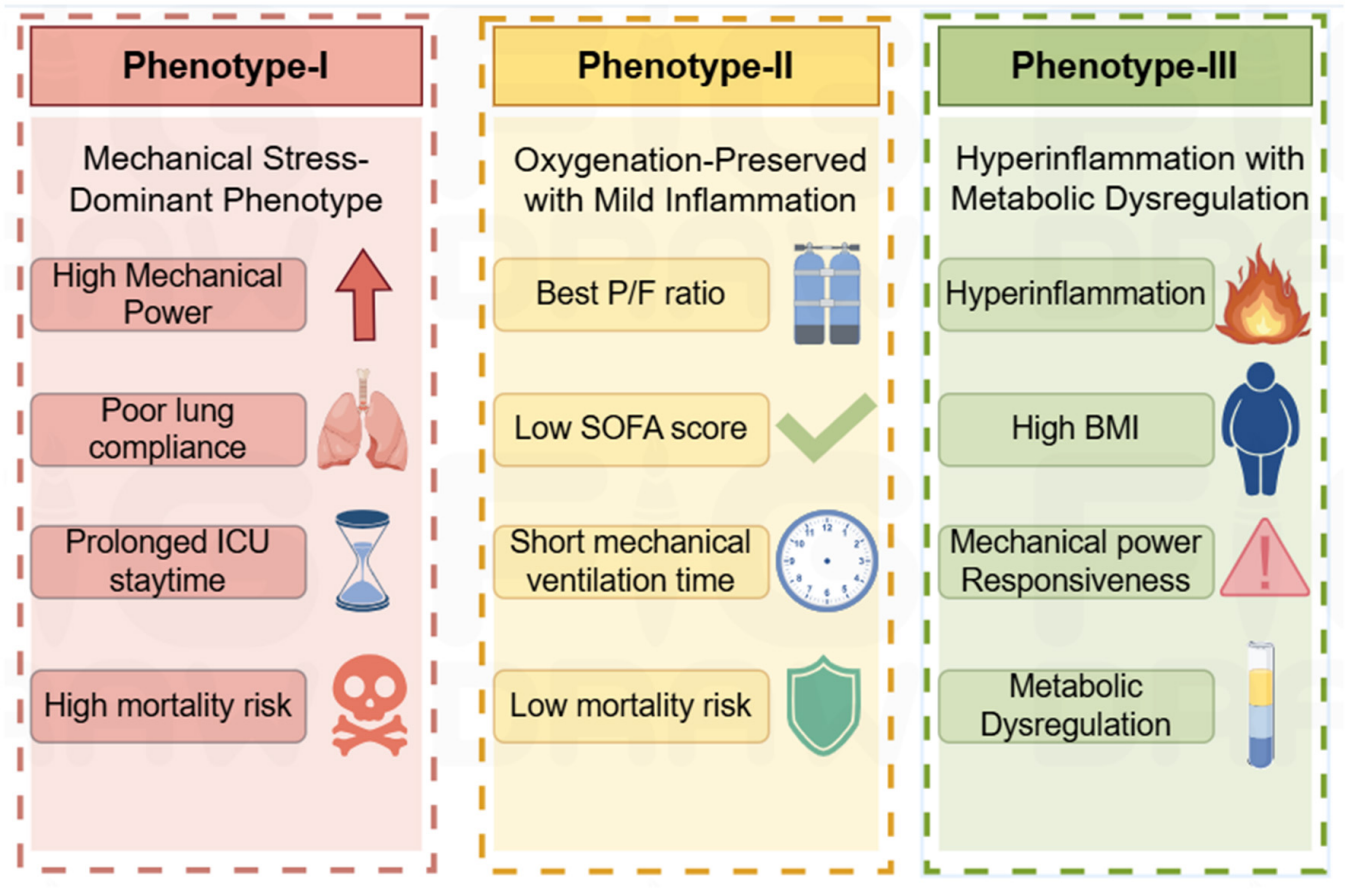
Graphical abstract of the phenotypes. This figure was created using Figdraw.

Consensus clustering analysis identified three distinct ARDS phenotypes. The cumulative distribution function (CDF), delta area curves, consensus matrices, and stability metrics consistently supported k = 3 as the optimal number of clusters (Figure 4; Additional File Figures S6–S8). Clinical characteristics of the three phenotypes are summarized in Additional File Table S3 and illustrated using line plots and t-SNE visualization (Additional File Figures S9, S10; Figure 5). Phenotype I (*n* = 483) exhibited the highest 28-day mortality (29%) and was characterized by poor lung compliance, higher respiratory rates, elevated plateau and peak pressures, and prolonged ICU stays. Phenotype II (*n* = 434) demonstrated the best oxygenation, lowest SOFA scores, shortest duration of mechanical ventilation, and the lowest mortality (20.5%). Phenotype III (*n* = 416) was characterized by higher BMI, preserved lung compliance, and pronounced systemic metabolic and inflammatory abnormalities, including elevated white blood cell counts, creatinine, and lactate levels.

### Heterogeneity in the association between mechanical power and outcomes

**Figure 6:**
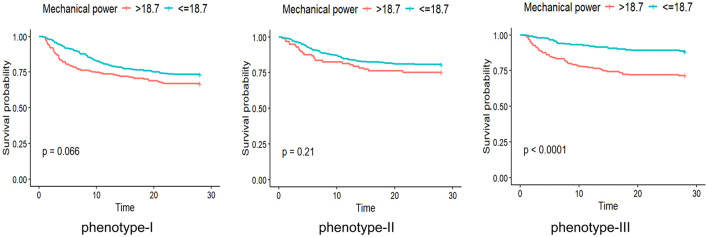
Association of mechanical power with mortality across ARDS phenotypes. Kaplan–Meier survival curves illustrating the survival probability for ARDS patients grouped by a mechanical power (MP) cutoff of 18.7 J/min.

The association between mechanical power and outcomes differed significantly across phenotypes. In Phenotype III, higher MP was consistently associated with increased 28-day mortality, in-hospital mortality, and ICU mortality (all *P* < 0.05). In Phenotype I, MP was significantly associated with ICU and in-hospital mortality but not 28-day mortality. No significant association between MP and mortality was observed in Phenotype II (Table 2). When stratified by the predefined cutoff of 18.7 J/min, patients with high MP experienced longer mechanical ventilation duration, prolonged ICU stays, and higher mortality, particularly in Phenotype III. In contrast, MP stratification did not significantly influence mortality outcomes in Phenotype II (Figure 6; Table 3).

**Table 2:** The relationship between mechanical power and prognosis in difference phenotypes.

**Variable**	**Phenotype**	**Odds ratio**	**Lower bound 95% CI**	**Upper bound 95% CI**	***p*-value**
28 d mortality	Phenotype-I	1.027	1.000	1.056	0.050
	Phenotype-II	1.019	0.981	1.058	0.334
	Phenotype-III	1.050	1.018	1.083	0.002
Hospital mortality	Phenotype-I	1.028	1.001	1.056	0.042
	Phenotype-II	1.009	0.972	1.047	0.635
	Phenotype-III	1.053	1.021	1.085	0.001
ICU mortality	Phenotype-I	1.031	1.003	1.060	0.030
	Phenotype-II	1.034	0.994	1.076	0.098
	Phenotype-III	1.057	1.023	1.091	0.001

**Table 3:** The clinical characteristics for each phenotype, comparing patients with MP ≤ 18.7 J/min and MP > 18.7 J/min, with statistical significance for mechanical ventilation hours, ICU stays, 28-day mortality, hospital mortality, and ICU mortality.

	**Phenotype-I**			**Phenotype-II**			**Phenotype-III**		
	**MP** < = **18.7 (*****n*** = **293)**	**MP** > **18.7 (*****n*** = **190)**	***p***	**MP** < = **18.7 (*****n*** = **337)**	**MP** > **18.7 (*****n*** = **97)**	***p***	**MP** < = **18.7 (*****n*** = **202)**	**MP** > **18.7 (*****n*** = **214)**	***p***
Mechanical ventilation hour [median (IQR)]	61.50 [37.00, 118.00]	99.00 [59.00, 166.50]	< 0.001	50.00 [33.00, 78.00]	64.00 [43.00, 95.00]	0.004	53.80 [35.78, 98.17]	75.50 [43.55, 119.88]	0.001
ICU staydays [median (IQR)]	8.68 [4.75, 13.56]	11.13 [6.10, 17.41]	0.002	7.55 [4.42, 13.81]	8.58 [5.61, 12.90]	0.347	8.16 [5.05, 13.96]	10.05 [5.46, 15.96]	0.241
28 d mortality (%)	78 (26.62)	63 (33.16)	0.150	65 (19.29)	24 (24.74)	0.303	24 (11.88)	61 (28.50)	< 0.001
Hospital mortality (%)	80 (27.30)	66 (34.74)	0.102	71 (21.07)	25 (25.77)	0.398	26 (12.87)	66 (30.84)	< 0.001
ICU mortality (%)	67 (22.87)	60 (31.58)	0.044	49 (14.54)	22 (22.68)	0.079	21 (10.40)	57 (26.64)	< 0.001

### External validation

In the external validation cohort, logistic regression based on in-hospital mortality demonstrated that increasing mechanical power was significantly associated with a higher risk of death (OR = 1.203, 95% CI 1.058–1.367; *P* = 0.005). Consistently, time-to-event survival analysis further confirmed that higher mechanical power was associated with reduced in-hospital survival (HR = 1.143, 95% CI 1.063–1.229; *P* < 0.001). Subsequently, patients were stratified using the 18.7 J/min cutoff derived from the MIMIC-IV cohort, and Kaplan–Meier analysis showed that the high-MP group had significantly lower in-hospital survival compared with the low-MP group (log-rank *P* = 0.0028; Additional file Figure S11). These findings collectively reinforce the robustness and external applicability of mechanical power as a prognostic indicator in ARDS patients receiving invasive mechanical ventilation. Notably, in the external validation cohort, the survival probability of the high mechanical power group declined to zero during hospitalization (Additional file Figure S11), reflecting that all 19 patients in this subgroup experienced in-hospital death. This extreme pattern is likely influenced by the small sample size and high baseline risk of the high-MP group, and therefore should be interpreted with caution.

### Sensitivity analysis

To further evaluate whether the association between mechanical power and mortality was influenced by BMI-related mechanical characteristics, a sensitivity analysis was performed after excluding patients with high BMI values. The results remained highly consistent with the primary findings. Mechanical power continued to show a significant association with adverse outcomes, including 28-day mortality (OR = 1.051, 95% CI: 1.026–1.077; *P* < 0.001), in-hospital mortality (OR = 1.054, 95% CI: 1.029–1.079; *P* < 0.001), and ICU mortality (OR = 1.061, 95% CI: 1.035–1.089; *P* < 0.001). Mechanical power was also additionally analyzed as a categorical variable using the same cutoff value of 18.7 J/min. Consistent with the primary categorical analysis, patients in the high MP group exhibited significantly higher mortality than those in the low MP group, including 28-day mortality (OR = 2.097, 95% CI: 1.494–2.943; *P* < 0.001), ICU mortality (OR = 2.391, 95% CI: 1.680–3.403; *P* < 0.001), and in-hospital mortality (OR = 2.205, 95% CI: 1.580–3.078; *P* < 0.001). These results suggest that the association between mechanical power and prognosis remains consistent, and is unlikely to be explained solely by BMI-related physiological differences.

To further address the potential influence of spontaneous breathing on mechanical power estimation, we conducted an additional sensitivity analysis restricted to patients without spontaneous respiratory. In this subgroup, patients were stratified according to the previously identified mechanical power cutoff value of 18.7 J/min (Additional file Figure S12). In the unadjusted analysis, patients with high mechanical power (MP > 18.7 J/min) exhibited significantly higher mortality compared with those with low mechanical power (MP ≤ 18.7 J/min), including 28-day mortality (OR = 1.911, 95% CI: 1.368–2.669; *P* < 0.001), ICU mortality (OR = 2.123, 95% CI: 1.495–3.015; *P* < 0.001), and in-hospital mortality (OR = 1.854, 95% CI: 1.337–2.572; *P* < 0.001). After propensity score matching (*N* = 239 per group; Additional file Table S4), the association between high mechanical power and increased mortality remained statistically significant. High mechanical power was associated with higher 28-day mortality (OR = 1.645, 95% CI: 1.069–2.530; *P* = 0.024), ICU mortality (OR = 1.628, 95% CI: 1.042–2.542; *P* = 0.032), and in-hospital mortality (OR = 1.615, 95% CI: 1.059–2.464; *P* = 0.026) (Additional file Figure S13). Consistently, survival analysis demonstrated a significantly higher risk of death in the high mechanical power group compared with the low mechanical power group (log-rank *P* = 0.0015) (Additional file Figure S14). Together, these findings indicate that the association between elevated mechanical power and adverse outcomes persists even when restricting the analysis to patients without spontaneous breathing, further supporting the robustness of our results.

## Discussion

This retrospective study investigated the association between mechanical power (MP) and clinical outcomes in patients with ARDS and explored whether this association differed across data-driven ARDS phenotypes. Three principal findings emerged. First, higher mechanical power within the first 24 h after ARDS diagnosis was significantly associated with increased ICU, in-hospital, and 28-day mortality. Second, among the components of MP, the elastic–dynamic component—closely related to driving pressure and respiratory rate—demonstrated the strongest association with mortality. Third, distinct ARDS phenotypes exhibited heterogeneous susceptibility to elevated mechanical power, suggesting that the prognostic association of MP is not uniform across patients.

### Mechanical power and mortality

Mechanical power integrates multiple ventilatory parameters into a single metric reflecting the total mechanical power delivered to the respiratory system (18). Compared with individual ventilator variables, MP provides a more comprehensive representation of the mechanical stress imposed on injured lungs. In this study, higher early MP was independently associated with worse short-term outcomes, even after propensity score matching and external validation, supporting MP as a robust marker of ventilator-associated risk.

The MP cutoff of 18.7 J/min identified in this study should be interpreted as a physiological risk threshold rather than a definitive prognostic boundary. ARDS outcomes are influenced by multiple interacting factors, and MP should be viewed as a modifiable contributor to ventilator-induced lung injury (VILI), rather than a standalone predictor of mortality. We focused on MP measured during the first 24 h after ARDS diagnosis because early ventilatory exposure is clinically actionable and may influence subsequent disease trajectory.

### Components of mechanical power and mortality

Mechanical power is a composite measure that incorporates resistive, static, and dynamic (elastic) forces, all of which contribute to tissue strain during mechanical ventilation (15). However, the relative contribution of each mechanical power component to outcomes associated with ventilator-induced lung injury remains uncertain, which may limit the interpretability of elevated MP in clinical settings. In our study, we analyzed MP components and found that the elastic-dynamic component was most strongly associated with mortality, consistent with Costa et al. (15). This suggests that clinicians should focus on adjusting variables that contribute to the elastic-dynamic component (such as driving pressure and respiratory rate) when mechanical power is high. Furthermore, we explored the association between individual ventilator parameters and mortality. Our findings indicate that respiratory rate had the strongest associations with mortality. This stronger link between the elastic-dynamic component and respiratory rate aligns with physiologic theories, as cyclic over distension and repetitive mechanical stress are likely more injurious and drive ventilator-induced lung injury (VILI) more than resistive work alone. These findings highlight the significance of certain ventilatory parameters—especially driving pressure and respiratory rate—in guiding therapeutic decisions, which can help clinicians optimize ventilation strategies and reduce mortality in ARDS patients. The same result could be found in the study guided by Tonna et al. (19, 20) that demonstrated mechanical power is not solely dependent on tidal volume and driving pressure, but also significantly affected by respiratory rate. Overall, these results support that MP is a promising integrated metric to inform ventilator settings, and careful attention to respiratory rate and the dynamic elastic load may help reduce ventilator-induced harm. Given the strong association between the elastic-dynamic component, respiratory rate, and mortality, these parameters may represent important targets when MP is elevated. However, observational study cannot quantify the degree of improvement in outcomes—such as reductions in mortality or ICU stay—that could result from adjusting these variables. Prospective interventional trials are needed to determine the clinical benefit.

### Clinical relevance and stability of ARDS phenotypes

Using consensus clustering, we identified three clinically distinct ARDS phenotypes characterized by differences in respiratory mechanics, systemic inflammation, and outcomes. Importantly, the association between mechanical power and mortality varied across phenotypes. Patients with the Systemic Hyperinflammation phenotype showed the strongest association between elevated mechanical power and mortality, whereas no significant association between mechanical power and mortality was observed in the Oxygenation-Preserved phenotype.

These findings highlight the biological and clinical heterogeneity of ARDS and suggest that the prognostic relevance of mechanical power may depend on underlying pathophysiological context. Although clustering stability was supported by multiple internal validation metrics, including CDF curves, consensus matrices, intracluster consistency scores, and PAC values, these phenotypes should be considered exploratory and hypothesis-generating. External validation in independent cohorts is required to confirm their reproducibility and clinical utility.

### Clinical implications

Our results support a more individualized approach to mechanical ventilation in ARDS. Mechanical power may serve as a practical framework to integrate ventilatory parameters, with particular attention to driving pressure and respiratory rate. Moreover, phenotype-specific susceptibility to mechanical power suggests that certain patient subgroups—especially those with systemic hyperinflammation—may benefit most from stricter limitation of ventilatory energy load.

### Limitations

Several limitations should be acknowledged. First, the retrospective design precludes causal inference, and residual confounding cannot be excluded despite adjustment and propensity score matching. Second, mechanical power was calculated using ventilatory parameters from the first 24 h after ARDS diagnosis, which may not capture temporal variability. Third, intrinsic PEEP could not be reliably assessed, potentially influencing driving pressure and MP estimation. Fourth, data-driven phenotyping is sensitive to variable selection and preprocessing, and the identified phenotypes require external validation. Finally, because this study was not designed as a prediction model, discrimination and calibration metrics were not evaluated, and MP should not be interpreted as a standalone prognostic tool.

## Conclusion

In summary, higher mechanical power was independently associated with increased mortality in invasively ventilated ARDS patients, with the elastic dynamic component showing the strongest link to adverse outcomes. An MP threshold of approximately 18.7 J/min may indicate increased risk. The three data-driven ARDS phenotypes demonstrated heterogeneous responses to MP, underscoring the potential value of phenotype-guided ventilation strategies. Prospective studies are needed to confirm these findings and to determine their applicability in broader clinical settings.

## Data Availability

Publicly available datasets were analyzed in this study. This data can be found here: All data and material were available at https://physionet.org/content/mimiciv/2.2/.
